# The Impact of Bioinformatics Pipelines on Microbiota Studies: Does the Analytical “Microscope” Affect the Biological Interpretation?

**DOI:** 10.3390/microorganisms7100393

**Published:** 2019-09-26

**Authors:** Léa Siegwald, Ségolène Caboche, Gaël Even, Eric Viscogliosi, Christophe Audebert, Magali Chabé

**Affiliations:** 1 PEGASE-Biosciences (Plateforme d’Expertises Génomiques Appliquées aux Sciences Expérimentales), Institut Pasteur de Lille, 59000 Lille, France; lea@siegwald.net (L.S.); segolene.caboche@univ-lille.fr (S.C.); g.even@genesdiffusion.com (G.E.); c.audebert@genesdiffusion.com (C.A.); 2 Université Lille, CNRS, Inserm, CHU Lille, Institut Pasteur de Lille, U1019–UMR 8204–CIIL–Centre d’Infection et d’Immunité de Lille, 59000 Lille, France; eric.viscogliosi@pasteur-lille.fr; 3 Gènes Diffusion, 3595, route de Tournai, 59501 Douai, France

**Keywords:** 16S targeted metagenomics, metagenetics, bioinformatics pipelines, human gut microbiota, case-control study

## Abstract

Targeted metagenomics is the solution of choice to reveal differential microbial profiles (defined by richness, diversity and composition) as part of case-control studies. It is well documented that each data processing step may have the potential to introduce bias in the results. However, selecting a bioinformatics pipeline to analyze high-throughput sequencing data from A to Z remains one of the critical considerations in a case-control microbiota study design. Consequently, the aim of this study was to assess whether the same biological conclusions regarding human gut microbiota composition and diversity could be reached using different bioinformatics pipelines. In this work, we considered four pipelines (mothur, QIIME, kraken and CLARK) with different versions and databases, and examined their impact on the outcome of metagenetic analysis of Ion Torrent 16S sequencing data. We re-analyzed a case-control study evaluating the impact of the colonization of the intestinal protozoa *Blastocystis* sp. on the human gut microbial profile. Although most pipelines reported the same trends in this case-control study, we demonstrated how the use of different pipelines affects the biological conclusions that can be drawn. Targeted metagenomics must therefore rather be considered as a profiling tool to obtain a broad sense of the variations of the microbiota, rather than an accurate identification tool.

## 1. Introduction

Metagenetics (i.e., targeted metagenomics) is the favored solution for comparing different microbial profiles as part of case-control studies. Indeed, 16S rRNA gene amplicon sequencing of a large amount of samples in different homogeneous groups, defined by different clinical and/or environmental factors, allows multivariate analyses to evaluate the alterations of microbiota (defined by richness, diversity and composition) between those groups. Such studies usually follow sampling recommendations made towards robust experimental designs to limit experimental variation (by using homogeneous samples in the same group and sufficient group sizes). Moreover, characterizing microbial communities via next-generation sequencing is subject to a number of pitfalls in the whole metagenomics process workflow. It could be mentioned: (i) biological biases like sampling processes, storage, handling and DNA extraction; (ii) biotechnological biases that occur between DNA extraction and sequence reads obtaining and that are related to DNA amplification and sequencing (such as the choice of target amplicon); and (iii) analytical biases until the taxonomic assignments of the sequencing reads have been achieved. Biological and biotechnical biases are relatively well described and sometimes difficult to control [1,2,3]. Likewise, robust statistical tools are used to interpret the results and extract relevant biological features, and a lot of warnings have been raised around their interpretation [4,5]. However, the biases least assessed of this whole process are induced by its analytical core meaning converting raw sequences into microbial profiles.

A lot of analytical approaches are available, and each analytical pipeline (defined by a succession of analytical steps and a reference database) can be affected by different variables of the experimental design. Such variations can lead to different results depending on which pipeline is used, as we showed in a previous study using simulated data [6]. For example, different analytical pipelines are more or less able to handle sequencing errors, which can alter the outcoming microbial profile of a sample. Sequencing errors are still a problem with second generation benchtop sequencers, which do unfortunately generate noisy data. The Ion Torrent PGM generates sequencing errors for which no specific denoising nor error-correction algorithm exists; likewise, the Illumina MiSeq has been reported to generate low quality sequences at the end of longer reads [7]. New analytical approaches for targeted metagenomics data have recently emerged and are based on error correction of reads to discriminate between real variations, at the single nucleotide level, and sequencing errors. These methods, such as Dada 2 [8] and Deblur [9] allow a most accurate assignment of reads to be obtained and to reach a better taxonomic resolution and are now integrated in the version 2 of the QIIME pipeline. However, Deblur2 is specific to Illumina technology (based on Illumina error profiles) and DADA2 has never been validated on Ion Torrent data. To our knowledge, no study has yet assessed the impact of analysis pipelines dealing with Ion Torrent targeted metagenomics data of a real comparative study in humans. It is therefore relevant to examine whether the choice of metagenomic analysis pipeline impacts the results of a real case-control study. In other words, could different analytical pipelines lead to a change of paradigm in the biological conclusions associated to the differential observations between case and control? 

To answer those questions, we evaluated four different analytical pipelines (and version/database variation for some) on Ion Torrent PGM 16S rRNA gene amplicon data we obtained in a previous case-control study [10]. This initial study revealed that the colonization with *Blastocystis* sp. is linked to a higher diversity of the bacterial microbiota [10], which is usually associated with a healthy gut [11]. Moreover, *Blastocystis*-colonized patients do not show a microbial signature of gut microbiota dysbiosis generally linked with inflammatory metabolic or infectious diseases [10]. Interestingly, we observed significant differences in the results after re-analysis with the four pipelines. This work, which would also deserve to be carried out on Illumina sequencing data, led us to venture some cautions about the interpretations of the results of case-control studies in regard to the analytical process. 

## 2. Materials and Methods 

### 2.1. Samples 

Previously published datasets of a case-control study [12] evaluating the impact of the colonization of the intestinal protozoa *Blastocystis* sp. on the human gut microbial profile were used in this work. Briefly, a robust sampling protocol led to the selection of 96 patients, segregated into two groups: those colonized by the parasite, and those *Blastocystis*-free. Indeed, to avoid a bias in the selection of samples, statistical analyses were carried out on the clinical and environmental variables associated with each patient, in order to associate a risk score of colonization by *Blastocystis* sp. [10]. The 24 patients with the highest score and the 24 patients with the lowest score were selected from two different populations (i.e., positive or negative for *Blastocystis* sp.) for a total of 96 samples. The bacterial gut profile of those patients was studied by 16S rRNA targeted metagenomics sequencing on Ion Torrent PGM [10]. The raw datasets from each of the 96 sequenced samples were previously deposited into the Sequence Read Archive (SRA) of the NCBI, under Project ID PRJNA342805. The association between the sample name, sample ID (index), and *Blastocystis* sp. colonization status and group is available in Reference [12].

### 2.2. Bioinformatics Analysis

In the present work, all the sequencing data of this study were re-analyzed by varying only the primary analysis pipeline, defined by the software and advised database to convert reads into a taxa abundance table, also called the operation taxonomic unit (OTU) table, using the parameters by default advised by the pipelines developers. Two clustering-first pipelines (mothur 1.35.1 [13] with SILVA 119, and QIIME 1.9.0 [14] with Greengenes 13.8, named mothur and QIIME 1 respectively), and two assignment-first pipelines (kraken 2.0.7-β [15] with MiniKraken v1_8GB_201904_UPDATE, and CLARK 1.1.2 [16] with RefSeq 71 bacteria, named kraken mini and CLARK respectively) were used. To assess the impact of newly developed analytical approaches, we also used QIIME 2 with the DADA2 denoising method integrated in the qiime2 denoise-pyro script (named QIIME 2, altering the following parameter -p-trim-left 20 to fit IonTorrent technology as reported by users, followed up by default taxonomic assignment using qiime feature-classifier classify-sklearn on Greengenes 13.8). We also used kraken2 with the SILVA 132 database (named kraken SILVA), to evaluate the performance of an assignment-first pipeline with an amplicon-specific database. Since there is no standardized way to retrieve the full taxonomy of kraken2 assignments on SILVA, we manually used the kraken2 provided seqid2taxid.map and SILVA taxmap_ncbi dictionaries for that purpose. 

Of note, the 96 samples were processed in more than 24 h for mothur, one hour for QIIME 1 and QIIME 2, five minutes for CLARK and few seconds for kraken, confirming the significant disparities between the execution times of the different pipelines [6]. When needed, we used homemade scripts to convert the result of each pipeline to a global taxa abundance table for all samples. This table was interpreted using the same secondary analysis protocol as the original study to apply the same method of interpreting the results between all pipelines. Briefly, the singletons were first eliminated and each count table was then normalized by DeSeq2 [17]. 

### 2.3. Comparison of Bacterial Diversity, Richness and Composition and Statistical Analysis

To evaluate the impact of the analysis pipeline on the biological conclusions, we focused on two different microbiota features: the variation of bacterial richness and diversity between both groups of patients, and the variation of bacterial composition. 

For each sample, the richness (Chao1) and diversity indices (Shannon and Inverse Simpson) were calculated from the normalized counts, after taxonomic homogenization between databases and grouping at different taxonomic levels (order, family and genus), as was done in the original study [10]. The difference in these indices between the two groups of samples (patients colonized or not by *Blastocystis* sp.) was tested by a Mann-Whitney-Wilcoxon assay [18]. Moreover, Glass’s delta of Chao1 mean difference was calculated for each pipeline in order to measure the effect size of richness difference between the two groups of patients.

The normalized count tables were also interpreted for each pipeline by STAMP [19] to assess changes in taxa abundance at different taxonomic levels (order, family and genus). The representation chosen in STAMP to evaluate the results was the difference in the mean proportions of each taxon between the two groups of samples. Only taxa with an average difference of more than 1% were selected. For these taxa, the difference between the two groups was tested by a non-parametric Student test [20], with a Benjamini-Hochberg correction [21], such as used in the initial study [10].

## 3. Results and Discussion

### 3.1. Richness/Diversity

Richness and diversity measures can provide a first overview of the variations between case and control groups, by summarizing the microbial composition (richness), associated with the proportions of present taxa (diversity). Using these indices is not always obvious in a case-control study where one’s interest is directed towards the identification of significantly different taxa. However, the biological significance of those indices should not be overlooked. Indeed, in our context, richness and diversity are directly correlated with gut health [11,22] since loss of microbiota diversity appears as the most constant finding of intestinal dysbiosis [23].

Our previous work on simulated data showed that different analytical pipelines, especially in an error-prone sequencing context, do not estimate the same richness nor diversity for the same sample [6]. In the present work, we confirmed these results on real data. For example, mothur estimated a lower richness and diversity for the 96 samples analyzed (all groups combined) than the other pipelines (Chao1 and Shannon indices median (interquartile range (IQR)): 41 (12.54) and 5.05 (0.47) respectively). Kraken SILVA on the contrary was the pipeline estimating the highest richness and diversity (Chao1 and Shannon indices median (IQR): 130.8 (53.64), 6.42 (0.68) respectively). It was therefore legitimate to wonder whether this variation in absolute richness between pipelines had an impact on the biological conclusions drawn when comparing the two groups of patients.

**Figure 1:**
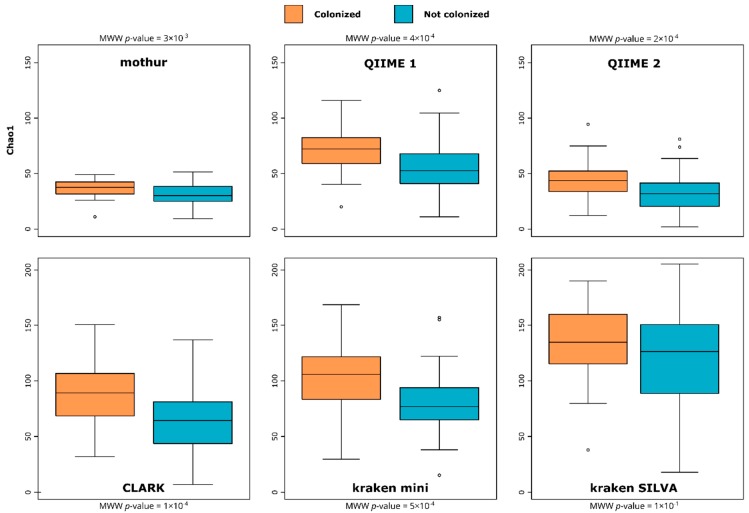
Chao1 indices boxplots at the family level between both groups of patients (*Blastocystis*-colonized and *Blastocystis*-free), for all pipelines. Difference between groups was tested using a Mann–Whitney-Wilcoxon (MWW) test [18,24].

Figure 1 represents how the variations of richness between *Blastocystis*-colonized and *Blastocystis*-free patients were interpreted when using different primary analytical pipelines. For all pipelines except kraken SILVA, Chao1 richness index was significantly higher for *Blastocystis*-colonized patients, concurring the initial findings [10]. Such conclusions were also reached by looking at the Shannon diversity index (Figure S1). However, although the pipelines still showed overall higher bacterial richness in patients colonized by *Blastocystis* sp., the magnitude of this difference in bacterial richness between the two groups of patients varies by pipeline. One could easily emphasize the impact of *Blastocystis* on bacterial richness when using CLARK (Glass’s delta = 0.85), whereas the same conclusions would be more moderated when using kraken SILVA (Glass’s delta = 0.33). Moreover, the variation of richness between pipelines can especially be observed on extreme values. For both diversity and richness, the extreme values measured in each group were highly variable from pipeline to pipeline (as seen in Figure 1 and Figure S1). Those differences from a pipeline to another reinforce the importance of a robust sampling protocol with a sufficient number of samples per group compared, to maximize intragroup samples homogeneity and to minimize the impact of outliers on the results.

### 3.2. Composition

**Figure 2:**
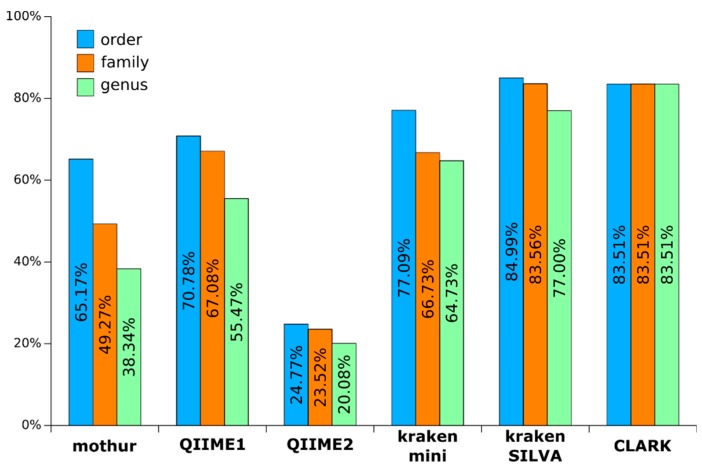
Assigned read proportions for each pipeline to different taxonomic levels (i.e., order, family and genus).

Metagenomics studies between case and control usually focus on the taxa whose reads are differentially abundant between both groups. Even if their abundance is biased by a lot of factors (PCR, gene number copy), such biases are assumed to happen equivalently in all samples. Therefore, a significant difference of abundance of taxa between two groups is assumed to be correlated with the biological phenomenon of interest. However, such differences are highly dependent on the analytical pipeline used. Indeed, one first difference observed in the results of the four pipelines evaluated was the important variation between the proportion of reads they were able to identify, meaning the proportion of original information represented in the results. For all samples studied in this work, mothur, QIIME 1, QIIME 2, kraken mini, kraken SILVA and CLARK were able to assign 49.27%, 67.08%, 23.52%, 66.73%, 83.56% and 83.51% of reads respectively, at the family level (Figure 2). This variation of recall directly impacts the estimation of composition of the samples, as we showed in our previous study [6].

This variation between pipelines can be explained by their inherent analytical philosophies. Some pipelines such as mothur and QIIME 2 favor the quality of data they use, at the expense of recall. Indeed, mothur recommends filtering the reads before clustering by aligning them on the database of reference, so that spurious unaligned reads are removed. The low recall of QIIME 2 can be explained by the default behavior of the integrated DADA2 algorithm, using a maxEE = 2 parameter. This allows for a maximum of two expected errors in the reads, a criterion which few of Ion Torrent reads comply to. Increasing the maxEE parameter to a value of 10 does indeed increase the assigned read proportions (to 72.65%, 69.70% and 59.74% at the order, family and genus level respectively). However, changing such a filtering parameter can also have a big impact on the precision of the results, and must be validated (on simulated Ion Torrent reads for example) before being advised as standard use.

Kraken assigns more reads when using SILVA than MiniKraken databases. Indeed, the use of an exhaustive amplicon-specific database allows for better discrimination between reads by preventing comparison of k-mers outside of the amplified region, and by increasing the number of discriminating k-mers in the database of reference.

**Figure 3:**
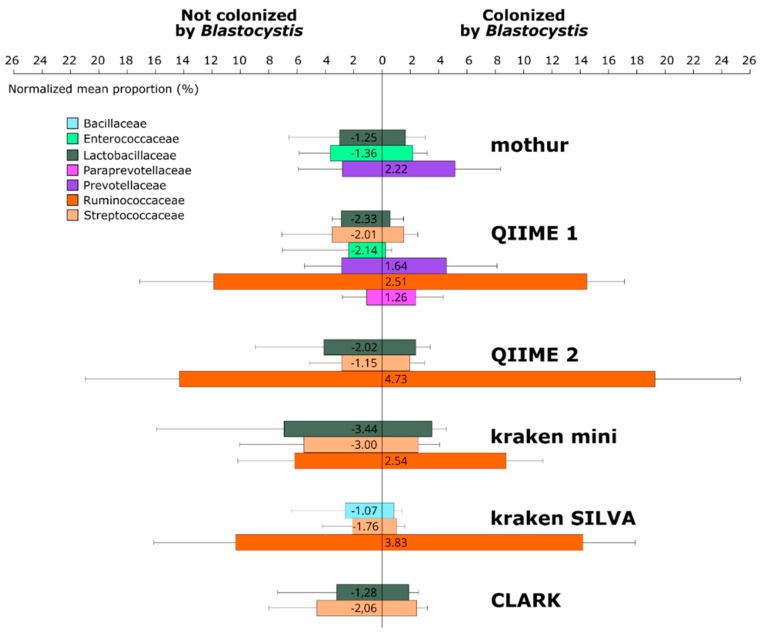
Mean proportions of each family are significantly different between both groups (the difference between groups has been tested reproducing the original study secondary analysis: using a non-parametric Student test with a Benjamini-Hochberg correction; only the families with a *p*-value < 0.05 and an effect size above 1% have been represented). Values on each bar is the difference of means between both groups.

When looking at the taxonomic composition of the samples generated by each pipeline, it is important to mention that no pipeline gave contradictory results: the significant difference of abundance of a taxon, if revealed by two or more pipelines, was always towards the same taxon. For example, in Figure 3, Lactobacillaceae, Enterococcaceae and Streptococcaceae were always more abundant in *Blastocystis*-free patients, whereas Prevotellaceae and Ruminococcaceae were always more abundant in *Blastocystis*-colonized ones, as described in the initial study.

However, by evaluating the differential abundance of taxa between *Blastocystis*-colonized and *Blastocystis*-free patients in the present work, discrepancies between pipelines were observed as soon as at the order level. In the initial study, two bacterial orders were considered significantly variable between the two groups of patients: Clostridiales were more abundant in patients colonized by the protozoa, while Lactobacillales were more abundant in non-colonized patients.

Here, only QIIME 1 and kraken mini revealed a significant difference in the Clostridiales order, more abundant in *Blastocystis*-colonized patients (6.69% and 5.91% respectively) (Figure S2). This order represents a lot of families whose abundance is directly correlated with a healthy gut (Ruminococcaceae, Eubacteriaceae, Lachnospiraceae [25]). Moreover, it is known that dysbiosis of the intestinal microbiota related to metabolic or infectious diseases like inflammatory bowel syndrome (IBS), inflammatory bowel disease (IBD) or enteric pathogens infections [26,27,28], that is commonly associated with inflammation of the lower gastro-intestinal tract, is typified by a reduction in bacterial diversity, a bloom of facultative anaerobic Gammaproteobacteria like Enterobacteriaceae and a decreased abundance of Clostridia [27,28]. Therefore, using QIIME 1 and kraken mini revealed in *Blastocystis*-colonized patients a significantly higher abundance of an order which could be essential in our gut health context. On the contrary, this information was not revealed by mothur, kraken SILVA, QIIME 2 and CLARK, which detect Clostridiales in both groups of samples, but whose variation between the two groups of patients was not significant enough to be represented. Still at the order level, all pipelines except kraken SILVA revealed a significantly more abundant Lactobacillales taxon for *Blastocystis*-free patients. Lactobacilli concentration (Gram+, Gram variable, facultative anaerobes) is generally decreased in irritable bowel syndrome (IBS) patients. Lactobacilli are generally considered nonpathogenic or even health-promoting. This observation shared by all pipelines can therefore be more reliable, but its relevance can still be questionable in regard to the magnitude of abundance variation. Indeed, the mean proportion of Lactobacillales was raised in *Blastocystis*-free patients from 4.09% for CLARK to 8.24% for mothur, not showing the same effect size (Figure S2).

The more detailed the taxonomic level, the more divergence between pipelines could be observed, concurring our previous findings on simulated datasets [6]. Indeed, we showed that precision and recall of all pipelines dropped when the taxonomic resolution was more detailed. In our case-control context, Figure 3 represents the differentially abundant taxa revealed by each pipeline at the family level.

In the initial study, *Blastocystis*-colonized patients exhibited a higher abundance of Ruminococcaceae and Prevotellaceae at the family level, whereas Lactobacillaceae, Enterococcaceae, Streptococcaceae and Enterobacteriaceae were enriched in *Blastocystis*-free patients. Of note, in this initial study, to analyze the Ion Torrent sequencing data, a homemade pipeline was developed using various publicly available tools such as Mothur [13] or EspritTree [29], databases such as the Silva small subunit RNA database and Ribosomal Database Project (RDP) and Perl/Python scripts [12].

In the present work, output analysis from QIIME 1 was the most closely related to the initial results. Conversely, other pipelines did not detect a significant difference between the taxa identified in the original study; however, the ones that were detected always varied in the same direction (e.g., *Lactobacillaceae* and *Streptococcaceae* were always more abundant in the *Blastocystis*-free group, and *Ruminococcaceae* in the *Blastocystis* colonized group). Regardless of the pipeline used in this study, Enterobacteriaceae, whose bloom is a marker of a dysbiotic intestine [30], were not found to be significantly different in the two patient groups. Concerning the families belonging to Clostridiales, mothur and CLARK did not reveal the higher abundance of Ruminococcaceae in *Blastocystis*-colonized patients. Lastly, a higher abundance of Prevotellaceae in *Blastocystis*-colonized patients was only revealed by QIIME 1 and mothur (Figure 3).

Furthermore, variations between pipelines were the most significant at the genus level (Figure S3). Indeed, QIIME 1 showed a variation profile close to the original study, whereas mothur displayed no significantly different genus between *Blastocystis*-colonized and *Blastocystis*-free patients. Kraken mini and CLARK concurred with QIIME 1 only for the *Lactobacillus* and *Streptococcus* taxa, more abundant in *Blastocystis*-free patients (Figure S3), while kraken SILVA and QIIME 2 showed distinct variation profiles. We speculate that those variations could be induced by the use of different databases and taxonomies, which directly impact the taxa classification. Indeed, classification and taxonomic assignment to a higher resolution remains a challenge for all of the pipelines, particularly in highly relevant orders like Enterobacteriales and Clostridiales. We presume duplicated, misannotated and imperfectly sequenced entries in reference databases contribute to classification errors. Further, an amplicon sequence can match multiple reference database entries with different taxonomic classifications, due to duplicated sequences and the amplicon region sequence being shared between distinct full-length sequences.

Kraken SILVA was the pipeline showing the most different results from the original study and all the other pipelines, despite its high recall (Section 3.2). A closer look at its results revealed that 56.46% of all the reads were assigned to uncharacterized reference sequences (annotated as “environmental samples”, “uncultured organism”, “ecological metagenome” or “organismal metagenome”). We already described in our previous study [6] how sensitive assignment-first pipelines are to the annotation and quality of the database of reference. While we believe that the use of an amplicon-specific database for such pipelines should improve their quality of results, we still lack a thoroughly curated and validated 16S rRNA gene database of reference that can be trustworthily used with assignment-first pipelines.

In light of such discrepancies, it seems difficult to assert robust biological conclusions on the nature of taxa differentially observed at the genus level. These conclusions, even supported by valid statistical analyses, can be inconsistent through different pipelines and lead to wrong inferences. Despite this fact, a lot of case-control studies in the literature focus on a detailed taxonomic level, like genus or even species, to draw biological conclusions, which can be perceived as more eloquent. For example, one would be tempted to highlight a variation of *Clostridium*, or even the pathogen *Clostridium difficile*, which will have much more impact in terms of associated biological conclusions than a variation of their related family Clostridiaceae, also containing many nonpathogenic taxa. This perception bias drives many studies to deepen their conclusions at the genus level, without moderating them in view of the lack of robustness of this kind of analysis.

To summarize, from the biologist’s perspective, the pipelines compared in this work did not really change the main biological conclusion of the initial study (i.e., *Blastocystis* colonization is not associated with dysbiosis generally observed in metabolic or infectious intestinal diseases). Indeed, in patients colonized by *Blastocystis*, no pipeline has found any bacterial families typically associated with dysbiosis associated with chronic inflammatory bowel diseases, characterized by an Enterobacteriaceae bloom and a decreased Clostridiales proportion. However, it is obvious that each bioinformatics microscope does not allow the biological object to be observed with the same focusing objective. In this sense, the more “objectives” are used, the more confidence can be placed in the picture.

## 4. Conclusions

Even if most pipelines reported the same trends in the present case-control study, we demonstrated how using different pipelines impacts the biological conclusions that can be drawn: whether it is for richness and diversity variation or differentially observed taxa. Particularly, we confirmed that richness and diversity indices cannot be interpreted in an absolute way, since they are far too variable from one pipeline to another. Also, these indices are good estimators of potential differences between two sample groups, since in our example, their variation between the two patient groups was confirmed regardless of the pipeline used, and even if the effect size of bacterial richness difference between the two groups was different according to the pipeline used.

The variation of biological conclusions we observed in this work with each pipeline was not related to the direction of the main biological message, but in the number and strength of observations made to support it. Like optical aberrations can affect the image seen through a microscope, analytical biases can impact the image of a given microbiota. One should therefore consider metagenetics as a profiling tool to get a broad sense of microbiota variations, rather than an exact identification tool.

Despite their appeal, new analytical approaches like denoising or more suited databases should never be used blindly without proper validation.

A first way to grasp the analytical bias in the results and to validate such approaches could be to use simulated datasets representative of the microbiota of interest, using an evaluation protocol like the one we propose in our previous study [6]. The analysis of such a dataset is cost-effective, could unveil potential analytical biases and help adjust default parameters. Another approach we recommend is the use of two different pipelines, in order to strengthen the conclusions by providing another viewpoint, using different databases and analytical concepts, to analyze the same dataset. For Ion Torrent datasets, we recommend using the validated standard QIIME 1 pipeline with confirmation of the results using kraken mini. While the integration of DADA2 in QIIME 2 has been shown to drastically improve the analyses results of Illumina data, it stills lacks proper evaluation and parameters optimization to be routinely used with Ion Torrent data at this time. In the same spirit, it can be tempting to use amplicon-specific databases with assignment-first pipelines. However, as confirmed by our results, one should always favor the quality of the database of reference over its specificity. Therefore, we still advise for now the use of RefSeq-based databases for k-mer based approaches, even for amplicon data. We hope that future curation of amplicon-specific databases will allow their use with assignment-first pipelines with as much or even better precision.

The demonstration of such variations between pipelines reinforces the importance of detailing the analytical methodology used in any case-control study, as well as explicitly defining the metrics and secondary analysis methods that have been used. For example, the definition of richness and diversity indices is not often straightforward. It is not seldom that the Simpson index, Simpson’s index of diversity and Simpson’s reciprocal index are often mixed up into the “Simpson diversity index“ name, while they do not rely on the same definition. Such confusion can alter the reproducibility of studies, since the indices used are seldom explicitly defined in the Materials and Methods sections.

Thus, when a biologist examines microbiota data, the question may not be so much to challenge the validity of his results (is the genus I identify really there?), but rather to interpret them in full consciousness (are the composition differences between groups confirmed by another pipeline?). In our opinion, it is the biologist’s responsibility to judge the robustness of the microscope(s) they use in order to obtain valid biological conclusions. Although this article focuses on the analytical part of the workflow of a metagenetics study, it should be noted that the experimental design is the first element that can ensure the robustness of this kind of analysis and prevent misinterpretation. However, unlike the experimental design, which is a “one-shot” operation, analytical methods can be multiple, so that on the same data set, it is possible and therefore even recommended to use several of them. It will then be important not to over-interpret results by choosing the most flattering analytical result.

## Supplementary Materials

Supplementary materials can be found at https://www.mdpi.com/2076-2607/7/10/393/s1.
