# Supplementary material for: The Impact of Bioinformatics Pipelines on Microbiota Studies: Does the Analytical “Microscope” Affect the Biological Interpretation?
Source: Microorganisms. 2019 Sep 26;7(10):393. doi: 10.3390/microorganisms7100393 (PMC6843237; doi:10.3390/microorganisms7100393)
Supplement: Supplementary file 1 [file microorganisms-07-00393-s001.pdf]

The impact of bioinformatics pipelines on microbiota studies: does the analytical "microscope" affect the biological interpretation?

Léa Siegwald[1], Ségolène Caboche[1,2], Gaël Even[1,3], Eric Viscogliosi[2], Christophe Audebert[1,3,#] and Magali Chabé[2, #,*]

<u>Suppl. File: Figure S1, Figure S2 & Figure S3</u>

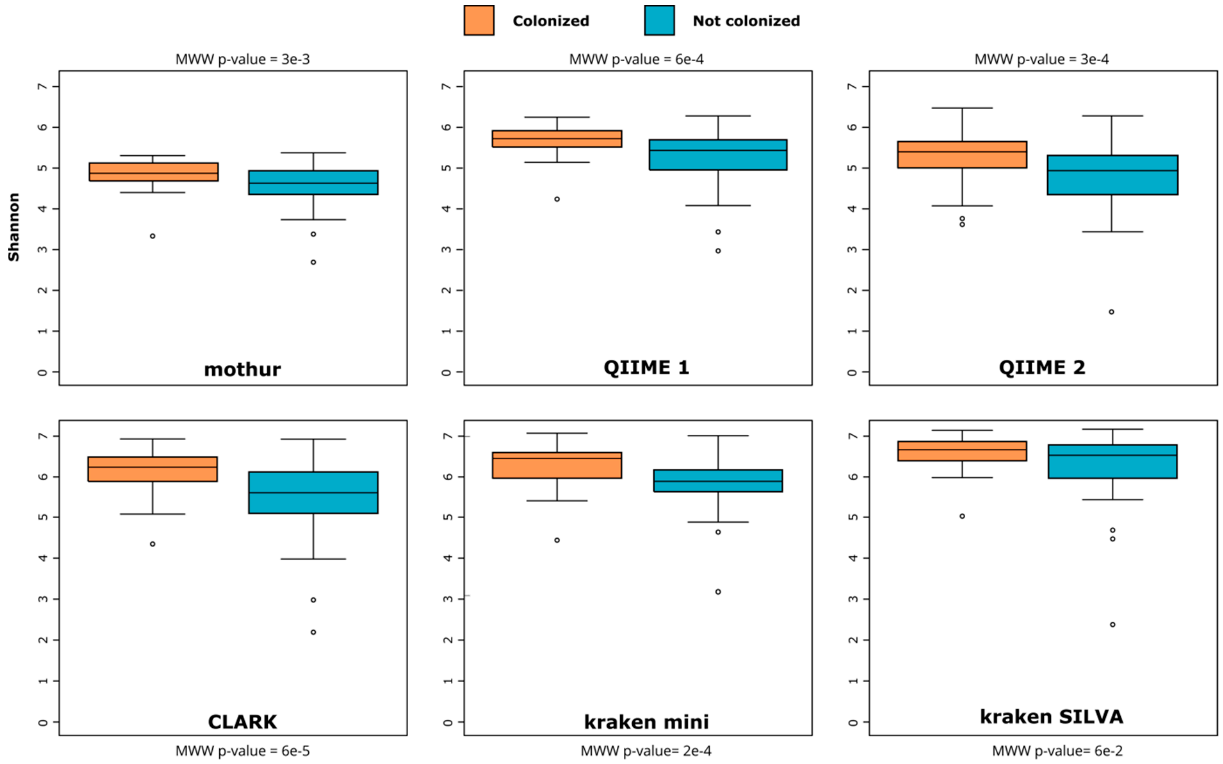

Figure S1. Shannon indices boxplots at the family level between both groups of patients (*Blastocystis*-colonized and *Blastocystis*-free), for all pipelines. Difference between groups has been tested using a Mann-Whitney-Wilcoxon (MWW) test [18,24].

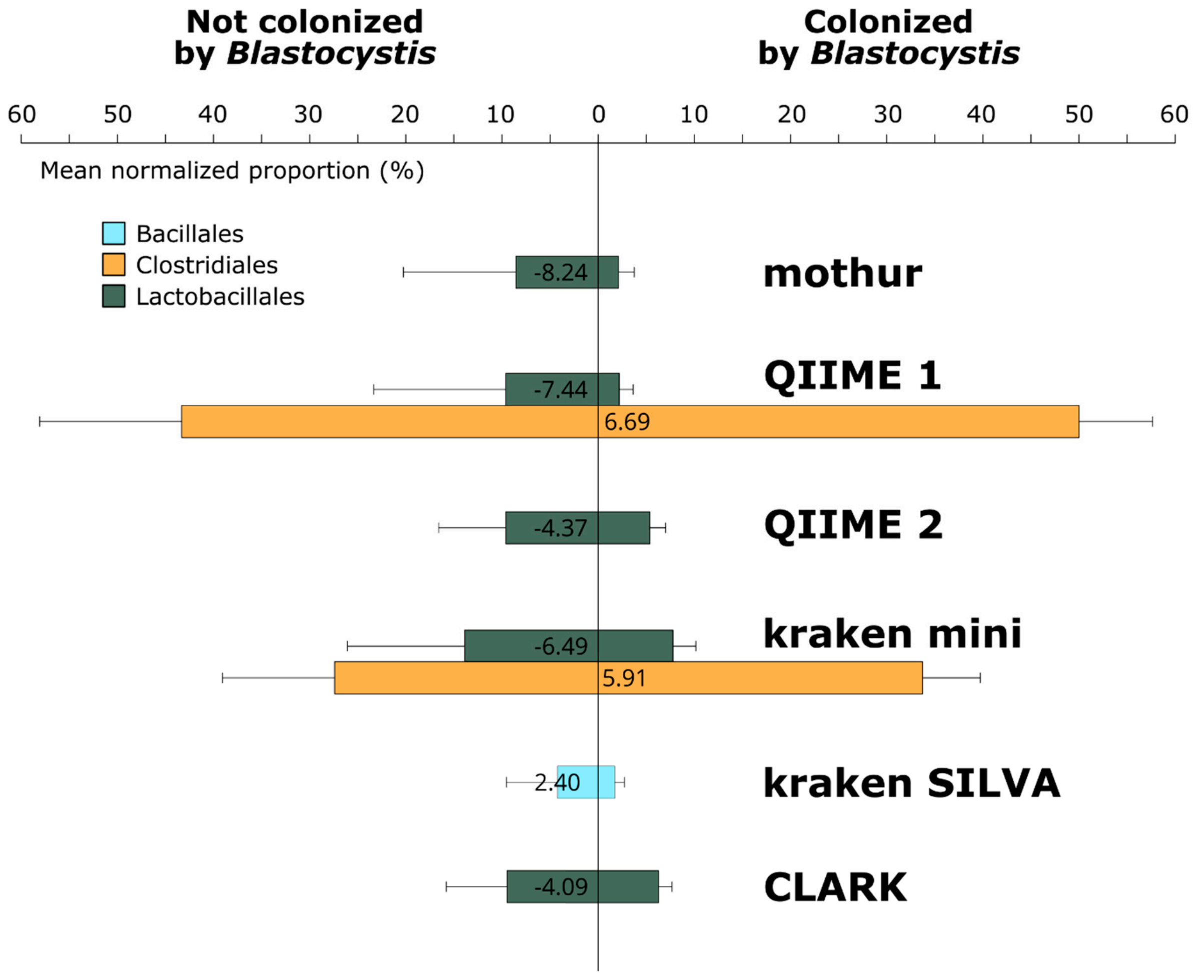

Figure S2. Mean proportions of each order significantly different between both groups (the difference between groups has been tested reproducing the original study secondary analysis: using a non-parametric Student test with a Benjamini-Hochberg correction; only the orders with a q-value < 0.05 and an effect size above 1 % have been represented). Values on each bar is the difference of means between both groups.

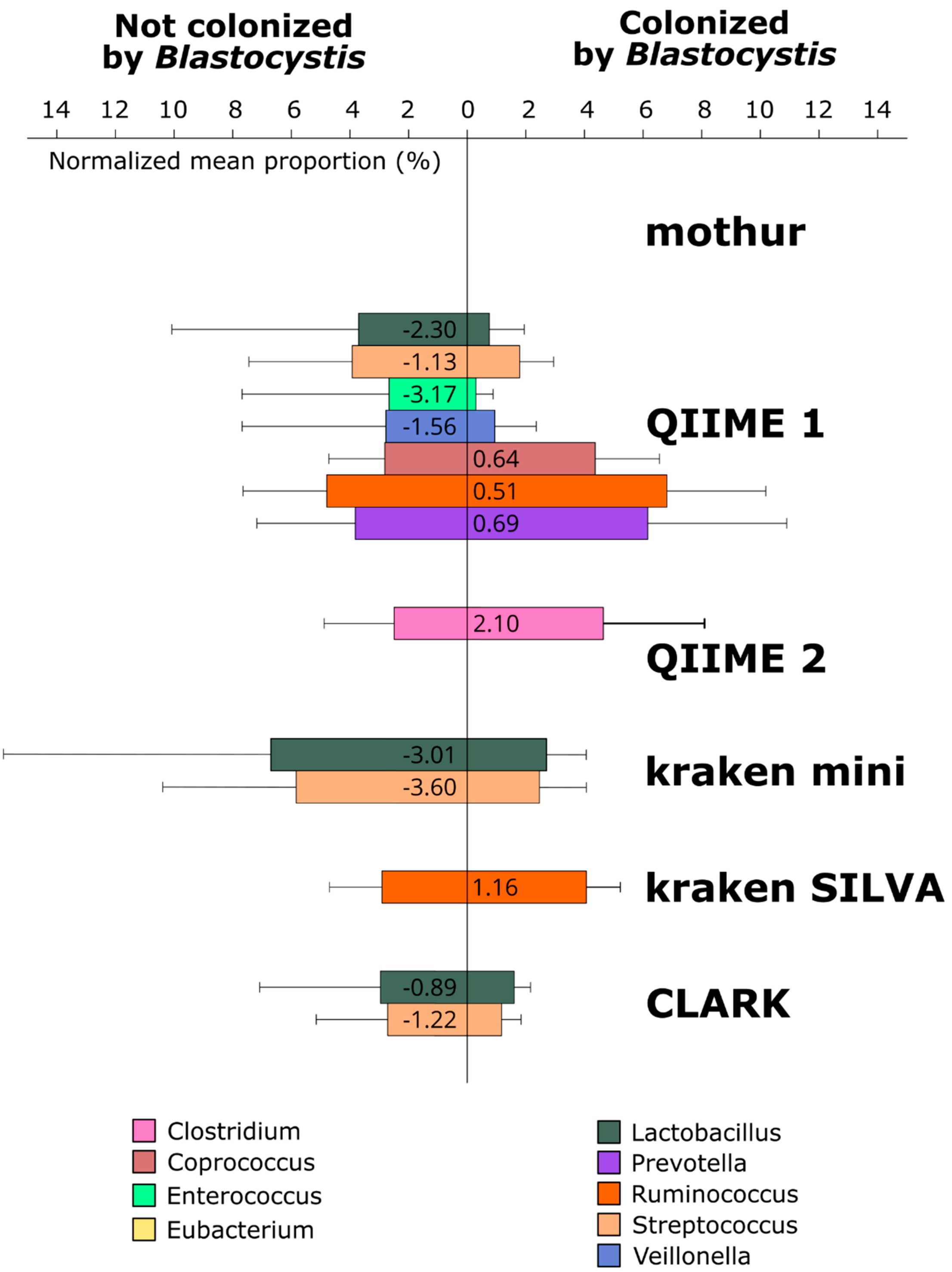

Figure S3. Mean proportions of each genus significantly different between both groups (the difference between groups has been tested reproducing the original study secondary analysis: using a non-parametric Student test with a Benjamini-Hochberg correction; only the genera with a q-value < 0.05 and an effect size above 1 % have been represented). Values on each bar is the difference of means between both groups.
